# Generalized Information Equilibrium Approaches to EEG Sleep Stage Discrimination

**DOI:** 10.1155/2016/6450126

**Published:** 2016-07-19

**Authors:** Todd Zorick, Jason Smith

**Affiliations:** ^1^Department of Psychiatry, Veterans Affairs Greater Los Angeles Healthcare System, Los Angeles, CA 90073, USA; ^2^Department of Psychiatry and Biobehavioral Sciences, UCLA, Los Angeles, CA, USA; ^3^The Boeing Company, Seattle, WA 98124, USA

## Abstract

Recent advances in neuroscience have raised the hypothesis that the underlying pattern of neuronal activation which results in electroencephalography (EEG) signals is via power-law distributed neuronal avalanches, while EEG signals are nonstationary. Therefore, spectral analysis of EEG may miss many properties inherent in such signals. A complete understanding of such dynamical systems requires knowledge of the underlying nonequilibrium thermodynamics. In recent work by Fielitz and Borchardt (2011, 2014), the concept of information equilibrium (IE) in information transfer processes has successfully characterized many different systems far from thermodynamic equilibrium. We utilized a publicly available database of polysomnogram EEG data from fourteen subjects with eight different one-minute tracings of sleep stage 2 and waking and an overlapping set of eleven subjects with eight different one-minute tracings of sleep stage 3. We applied principles of IE to model EEG as a system that transfers (equilibrates) information from the time domain to scalp-recorded voltages. We find that waking consciousness is readily distinguished from sleep stages 2 and 3 by several differences in mean information transfer constants. Principles of IE applied to EEG may therefore prove to be useful in the study of changes in brain function more generally.

## 1. Introduction

In electroencephalography (EEG), scalp electrodes measure electrical potential as a function of time [1]. EEG measures the sum of local field potentials in the region of cortex below the electrode, comprising ~10^9^ cortical neurons [1]. EEG is typically analyzed by spectral analysis (Fourier transform) that assesses power in frequency bands [1]. However, many studies over the last 20 years have demonstrated that the underlying cortical neuronal dynamics is nonlinear and that EEG signals are nonstationary (the mean and variance change over time unpredictably). This has been mostly convincingly demonstrated both* in vivo* and* in vitro* using multielectrode arrays on cortical tissue, demonstrating the presence of “neuronal avalanches” [2, 3].

Given that the cortical neuronal dynamics largely responsible for the summed local field potentials that comprise EEG are characterized by scale-free avalanches consistent with a system at a critical state that is well described by power-law dynamics, many attempts have been made to analyze EEG using methods derived from fractal and other nonlinear theories, with some degree of success [4–9]. Another avenue of physical understanding of cortical avalanche dynamics would be via statistical physics and thermodynamics; however, the relatively large magnitude changes in scalp-recorded voltages in EEG clearly could not be characteristic of a system in thermodynamic equilibrium [10]. Therefore, a thorough statistical physics understanding of EEG would involve a complete description of cortical nonequilibrium thermodynamics, which is not possible for a noninvasive technique such as EEG [10, 11]. Similarly, previously published information-theoretic shortcuts to a thermodynamic understanding (such as maximum entropy approaches) for EEG suffer from insufficient knowledge of appropriate constraints for microscopic variables [12, 13].

Instead, we propose to utilize the concept of generalized information transfer, where EEG could be modeled as an information transfer process [11, 14]. Generalized information equilibrium (IE) has been proposed as a system-independent mechanism to study systems far from thermodynamic equilibrium, with applications to astrophysics, economics, materials science, Newtonian physics, and thermodynamics [11, 14, 15]. The principles of IE were developed from Hartley's original description [16] of an amount of information (*I*):

(1) $$\begin{array}{c} I=K\cdot n, \end{array}$$

where *n* is the number of selected symbols and *K* = ln⁡*s* is a constant which depends on the number of symbols (*s*) available at each selection. Note that we use the natural logarithm, so that our natural information measure is in “nats” instead of “bits.” Following Fielitz and Borchardt (2014) we will use the Hartley definition of information to say that the information in a given process *x* is

(2) $$\begin{array}{c} I_{x}=K_{x}\cdot n_{x}\;\text{with }K_{x}=\ln s_{x}, \end{array}$$

where *s*_*x*_ is the size of the alphabet of symbols used to encode *x* and *n*_*x*_ is the number of symbols we select. A key assumption is that *n* ≫ 1 (which we have from the 10^8^ to 10^9^ neurons in the cortex underlying an electrode).

Note that the more commonly utilized Shannon entropy (*i*) defined as [17]

(3) $$\begin{array}{c} i=\frac{I}{n}=-\overset{s}{\underset{j=1}{\sum }}p_{j}\ln p_{j} \end{array}$$

reduces to the Hartley definition of information (*I*) when the probability of each symbol in the alphabet is equal (i.e., *p*_*j*_ is a constant). The use of Hartley's information theory, lacking any probabilistic assumptions, thus allows an estimation of information flow in any system even without access to knowledge of microscopic states or appropriate constraints in the case of maximum entropy approaches [11, 14]. It should also be noted here that Hartley information is a special case of the Rényi entropy for *α* = 0 [18]:

(4) $$\begin{array}{c} H_{\alpha }\left(\;X\;\right)=\frac{1}{1-\alpha }\ln\left(\;\overset{n}{\underset{i=1}{\sum }}p_{i}^{\alpha }\;\right). \end{array}$$

It has been demonstrated that one can use Hartley's information theory to define a natural amount of information for any system [11, 14]:

(5) $$\begin{array}{c} I_{x}=\kappa_{x}\frac{\left|\Delta x\right|}{\left|\delta x\right|}, \end{array}$$

where *κ*_*x*_ is the information transfer constant, |Δ*x*| is the absolute value, and |*δx*| is the signal of the process variable *x*, with |*δx* | ≪|Δ*x*|. Using this relationship, virtually any system where information flows from a source (*y*) to a destination (*x*) can be considered from the point of view of information transfer [11, 14]. The important point is, however, that the amount of information (*I*) must generally obey the inequality

(6) $$\begin{array}{c} I_{x}\leq I_{y}, \end{array}$$

when the process variable *x* is related to the information destination and the process variable *y* to the information source. For the current study, we assume ideal information transfer (*I*_*x*_ = *I*_*y*_) and, hence, information equilibrium (IE). Considering (5) one gets

(7) $$\begin{array}{c} \left(\;\frac{\left|\delta y\right|}{\left|\delta x\right|}\;\right)=\frac{K_{y}}{K_{x}}\frac{\left|\Delta y\right|}{\left|\Delta x\right|}. \end{array}$$

For convenience we will denote the ratio *κ*_*y*_/*κ*_*x*_ as *κ* and call it the information transfer constant for ideal information transfer or for IE. For EEG, we use (7) to define an information transfer constant (*κ*) for each time interval (Δ*t*) to the voltage reading (|Δ*V*|). We analyze the distribution of *κ* values to see if they are peaked around a well-defined mean. In that case we can interpret (7) (for small changes in the process variables *dx* and *dy*) as a differential equation:

(8) $$\begin{array}{c} \frac{d\left|\Delta V\right|}{d\left|\Delta t\right|}=\kappa_{avg}\frac{\left|\Delta V\right|}{\left|\Delta t\right|}, \end{array}$$

which has the solution

(9) $$\begin{array}{c} \left|\;\Delta V\;\right|\sim {\left|\;\Delta t\;\right|}^{\kappa_{\text{avg}}}. \end{array}$$

We will make a few observations here about the IE approach and its relationship to other physical descriptions of dynamic systems. For general information equilibrium, the solution to (8) can be rewritten as

(10) $$\begin{array}{c} {\left|\;\Delta V\;\right|}_{t_{0}+\Delta t}={\left|\;\Delta V\;\right|}_{t_{0}}\exp\left(\;\kappa \log\left(\;\frac{t_{0}+\Delta t}{t_{0}}\;\right)\;\right). \end{array}$$

Let us now set a new parameter, *λ* = *κt*_0_. Over short time scales (*t*_0_ ≫ Δ*t*), (10) reduces to

(11) $$\begin{array}{c} {\left|\;\Delta V\;\right|}_{t_{0}+\Delta t}={\left|\;\Delta V\;\right|}_{t_{0}}\exp\lambda \Delta t. \end{array}$$

Equation (11) is precisely the form of a Lyapunov exponent if the voltage measurement is considered as a superposition of a large number of neurons at different distances from the EEG sensor (i.e., |Δ*V*| is a sum over *n* individual neuron voltages near the sensor, mapping a* 4n* dimensional “phase space” to a voltage measurement (*ℝ* ^3^ × *V*)^*n*^→|*V*|). Lyapunov exponents are deeply related to the study of chaotic dynamical systems, with positive values indicating a chaotic system with exponential divergence from initial conditions [19]. For systems with power-law sensitivity to initial conditions, Lyapunov exponent analysis has been generalized to the scale-dependent Lyapunov exponent, which has been utilized to successfully describe many dynamic physical systems, including EEG-based seizure identification in humans (e.g., [5, 20–22]).

For the current study, we utilize a publicly available database of polysomnographic data for fourteen subjects with eight minutes each of waking and sleep stage 2 EEG (and eleven subjects with eight minutes of sleep stage 3 EEG) to assess for differences in patterns of *κ* values to assess the utility of IE in distinguishing different states of consciousness. Our hypothesis is that different states of consciousness can be identified by different distributions of *κ* and different *κ*_avg_ values.

## 2. Materials and Methods

### 2.1. Database

We utilized a publicly available EEG dataset (slpdb) http://www.physionet.org/, which was a polysomnogram study of patients with severe sleep apnea [23]. There were *n* = 14 subjects with 8 min of waking EEG and sleep stage 2 EEG and *n* = 11 subjects with 8 min of sleep stage 3 EEG. An additional dataset of *n* = 13 subjects of waking EEG, *n* = 10 subjects of REM sleep EEG, and *n* = 8 subjects of sleep stage 1 EEG (1 minute each, nonoverlapping with the larger 8 min EEG dataset) was also generated from the larger dataset. The exact dataset used has previously been described in a prior unrelated study [9]. EEG segments chosen for further analysis were selected on the basis of the absence of movement artifacts and disordered breathing, which limited the amount of suitable tracings. No demographic and limited clinical information was available from the dataset. Digitized 250 Hz EEG recordings on a 10–20 international system were used with a single EEG lead for each subject, which differed among subjects; no information was provided about reference electrode placement [9]. Use of the dataset for this study was approved by the VA West Los Angeles IRB.

### 2.2. *κ* Estimation

EEG is a time series of voltage readings *V*(*t*), where *t* = 1,2,…, *n* (length of series) for each value of *t* up to *n* − Δ*t*, given a time interval Δ*t*, so the *κ* values for each instant can be calculated:

(12) $$\begin{array}{c} \kappa_{\Delta t}=\frac{\log\left(\left|V_{t+\Delta t}-V_{t}\right|\right)}{log\left(\left|\Delta t\right|\right)}. \end{array}$$

Therefore, each segment of EEG would be characterized by a series of information transfer constant ratios, for different values of the time interval Δ*t* (i.e., 1, 2, 4, 8, time steps, etc.), and for each segment the mean *κ*_avg_ was calculated:

(13) $$\begin{array}{c} \kappa_{\text{avg}}=\frac{1}{n-\Delta t}\overset{n-\Delta t}{\underset{t=1}{\sum }}\kappa_{\Delta t}. \end{array}$$

Code for extracting *κ* values from EEG was written in R [24]. We used the natural log for transformation throughout. Values where *V*(*t*) = *V*(*t* + Δ*t*) were excluded from estimation (as the logarithm of zero is undefined).

### 2.3. Analyses

Probability density function (PDF) estimation was done using the R* density* package. Lomb-Scargle periodograms were done using the R package* cts* [25], designed to follow [26]. To assess statistically significant periodogram peaks, we utilized a *p* ≤ 0.01 threshold, heuristically estimating the maximum possible number of frequencies in the input PDF as twice the number of data points in the PDF [26]. All statistics were done in R [24]. For the REM sleep and sleep stage 1 dataset analysis with the reduced size dataset, we utilized generalized linear mixed modeling (GLMM) with unstructured covariance matrices to account for subject-specific effects, using the R package* nlme* [27].

## 3. Results

### 3.1. Waking Differs from Sleep Stages 2 and 3 in *κ* Values at Multiple Time Scales

**Figure 1:**
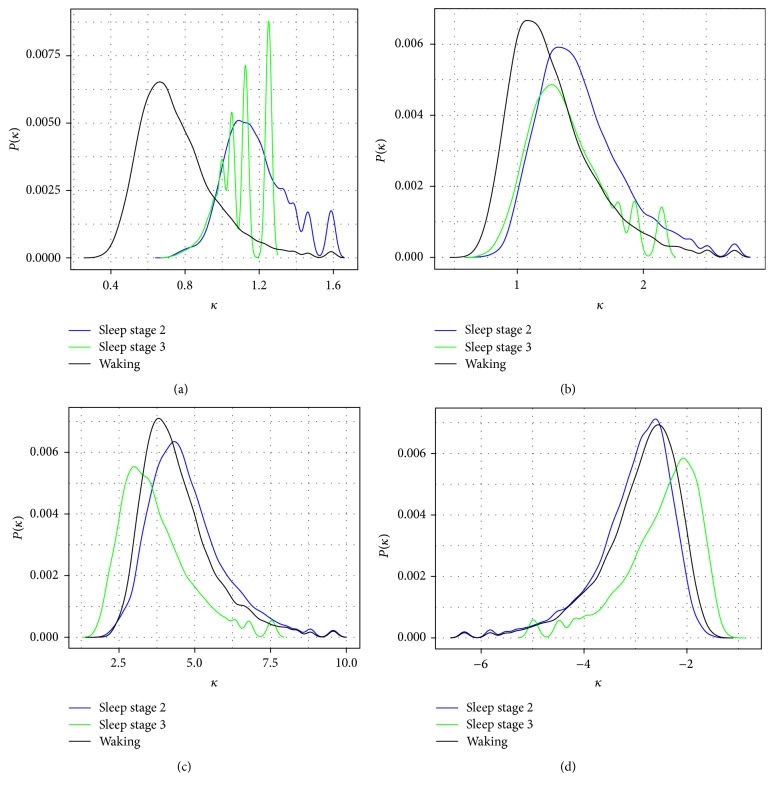
PDF estimations of *κ* values for one subject. One minute each of EEG from waking (black), sleep stage 2 (blue), and sleep stage 3 (green) from a single subject was analyzed for  *κ* values and the PDF estimated and plotted for each of (a) Δ*T* = 0.004 sec; (b) Δ*T* = 0.04 sec; (c) Δ*T* = 0.4 sec; (d) Δ*T* = 4 sec. *P*(*κ*): frequency of *κ* values. Note the characteristic amplitude fluctuations at all scales for the largest magnitude *κ* values.

We calculated the mean *κ* value for each segment in our database with a range of different Δ*T* values (0.004, 0.04, 0.4, and 4 seconds; Figure 1, Table 1). An example of the comparison in the PDFs of *κ* values for all three states of consciousness for 1 min each of EEG for a single subject at Δ*T* values from 0.004 to 4 sec is shown in Figure 1.

Segment-specific mean *κ* values were then analyzed by repeated-measures ANOVA with state of consciousness as the grouping variable and subject as the repeated measure (Table 1). These results demonstrate that waking EEG is clearly distinguishable from sleep stages 2 and 3 via segment mean *κ* values (Table 1). While waking and sleep stage 3 *κ* values differ strongly at 0.004-, 0.4-, and 4-second time scales, there is no difference between them at the 0.04-second time scale (Table 1). Interestingly, while there seems to be a pattern for waking and sleep stage 2 *κ* values to slowly become less different over time, if anything the opposite is true for the waking/sleep stage 3 comparison, where the 4-second time scale shows the largest difference between the two (comparing *F* values; Table 1).

### 3.2. Greater Proportion of Low Information Transfer at Δ*T* of 0.004 sec in Sleep Stages 2 and 3

Next, we assessed *κ* values for the proportion in each segment with values <0.2, as a heuristic indicator of low information transfer at the 250 Hz sampling rate (Table 2). Strikingly, both sleep stage 2 and (more so) sleep stage 3 have a greater proportion of low information transfer *κ* values than waking EEG (Table 2). At larger time steps, however, there was no difference between consciousness stages in the proportion of low information transfer (data not shown).

### 3.3. Waking Differs from Sleep Stages 2 and 3 in the Extent of Periodicity in the PDFs of *κ* Values at All Time Scales

**Figure 2:**
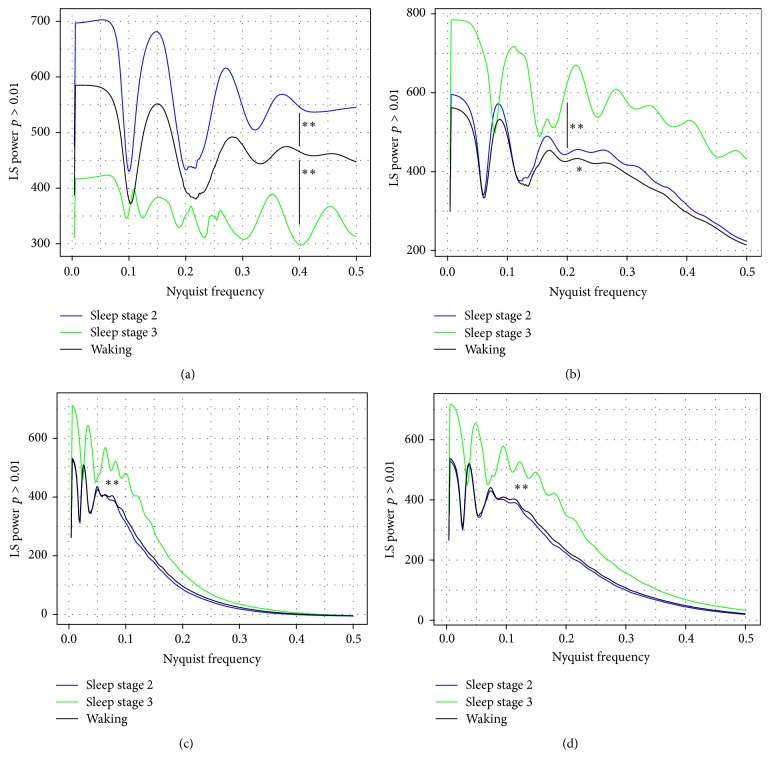
Lomb-Scargle periodogram power for *κ* value PDF estimations. Lines represent mean data for EEG of *n* = 14 subjects from waking (black) and sleep stage 2 (blue) and *n* = 11 subjects from sleep stage 3 (green), each with eight different one-minute segments. Data was analyzed for *κ* values and the PDF estimated and then normalized Lomb-Scargle periodogram area with a threshold of *p* > 0.01 calculated. (a) Δ*T* = 0.004 sec; (b) Δ*T* = 0.04 sec; (c) Δ*T* = 0.4 sec; (d) Δ*T* = 4 sec. ^**∗∗**^ *p* < 0.0001 by repeated-measures ANOVA for Lomb-Scargle periodogram area difference between waking and sleep stage 2 (a) and waking and sleep stage 3 (a–d); ^**∗**^ *p* < 0.01 between waking and sleep stage 2 (b).

As can be noted in Figure 1, there appears to be a periodicity in the PDF of *κ* values, in that certain magnitudes are enhanced, and others are diminished. In order to quantify this, we made PDF estimations for *κ* values for all segments and then performed a normalized Lomb-Scargle periodogram analysis, in order to assess for periodicity (Figure 2; Table 3). Sleep stage 2 exhibits enhanced periodicity, while sleep stage 3 shows diminished periodicity compared with waking at the 0.004 sec time step. For all other time steps, though, sleep stage 3 shows greater periodicity in the *κ* value PDF estimations than sleep stage 2 and waking consciousness (Figure 2; Table 3). Note that although sleep stage 2 and waking appear to be very similar at all other time steps (Figure 2), there are in fact modest quantitative differences between their periodicities (Table 3).

### 3.4. Waking Also Differs from Sleep Stage 1 and REM Sleep in Both Mean *κ* Values and the Extent of Periodicity in the PDFs of *κ* Values at Various Time Scales

Given the limitations of the physionet.org dataset, only a limited number of segments with artifact-free sleep stage 1 and REM sleep EEGs were available. As a further test of the IE description of EEG, we compared a nonoverlapping group of 1-minute tracings of waking, sleep stage 1, and REM sleep EEG by mean *κ* and Lomb-Scargle periodogram analysis of *κ* PDFs, using generalized linear mixed modeling (GLMM; Table 4). Neither sleep stage 1 nor REM sleep exhibited a significant proportion of low information transfer *κ* values at the sampling rate (data not shown). In terms of mean *κ* values, sleep stage 1 exhibits a larger value than waking only at the 250 Hz sampling rate, with only a trend towards significance at Δ*t* = 0.04 and 0.4 sec, and no difference at Δ*t* = 4 sec (Table 4). REM sleep mean *κ* values are larger than those for waking EEG at the sampling rate and Δ*t* = 0.04 sec, with only a trend at Δ*t* = 0.4 sec, with no difference between states at Δ*t* = 4 sec (Table 4).

Interestingly, both sleep stage 1 and REM sleep exhibited no difference from waking EEG in terms of the extent of periodicity in the *κ* value PDF estimations at the sampling rate, whereas both showed stronger differences with larger time steps (Table 4). Sleep stage 1 did not differ from waking in periodicity in the *κ* value PDF estimations at Δ*t* = 0.04 sec, demonstrated a trend towards an increase in periodicity at Δ*t* = 0.4 sec, and showed an increase in periodicity at Δ*t* = 4 sec (Table 4). By contrast, REM sleep exhibited a greater degree of periodicity in the *κ* value PDF estimations than waking at Δ*t* = 0.04, 0.4, and 4 sec.

## 4. Discussion

### 4.1. Generalized Information Theory and EEG

To our knowledge, this report is the first application of principles of IE to the study of EEG. Other information-theoretic approaches have a long history of neuroscience applications [28], but maximum entropy applications to EEG in particular have been limited by an appropriate understanding of microscopic constraints [12, 13]. The theoretical advantage of IE approaches to the analysis of EEG (or any other system) is that an explicit probabilistic understanding of the underlying system states (i.e., cortical local field potentials) is unnecessary; thus an estimation of “natural” information transfer can be assessed from a macro-observable (like time-dependent scalp voltage) alone [11, 14].

### 4.2. Utility of IE for the Study of Sleep Stage Discrimination

Our study demonstrates several interesting findings with regard to the application of IE to the analysis of polysomnogram data in EEG. Firstly, there is a clear distinction between waking, sleep stage 2, and sleep stage 3 consciousness in terms of mean *κ* values across different time scales (Table 1 [Table tab2] [Table tab3]), with sleep stage 1 and REM sleep consciousness having distinction at fewer time scales (Table 4). Secondly, waking differs from both sleep stage 2 and sleep stage 3 (but not sleep stage 1 or REM sleep) in terms of the proportion of low information transfer *κ* values at the sampling rate (250 Hz). Thirdly and perhaps most surprisingly, there is a clear periodicity of the PDF of the *κ* values at all time scales, which differs strongly between waking, sleep stage 2, and sleep stage 3, with more limited differences between waking and sleep stage 1 and REM sleep (Figure 2, Tables 3 and 4). Thus, there is an overall richness of the IE description of differences in sleep and consciousness states that may well suit it to be used as a general tool to study states of altered cortical function. Indeed, the apparent discriminative power of IE (Tables 1 [Table tab2] [Table tab3]–4) for sleep staging compares favorably with many other descriptions of computer-based analytic techniques for EEG, including fractals [6], multifractals [7, 9, 29, 30], and Tsallis entropy [4], not to mention automatic feature extraction from spectral analysis (reviewed in [31]).

### 4.3. Limitations

We utilized a publicly available dataset with minimal clinical or demographic information available. The number of subjects available was relatively small, and the number of EEG segments available was smaller still for sleep stage 1 and REM sleep. Waking consciousness is likely to be a heterogeneous state of brain activities; thus identifying this state only based upon clinical polysomnogram staging may limit the ability of any technique to assess for differences between waking and sleep stages. We can not exclude the possibility that some of the observed differences between states of consciousness were caused by differences in motor or muscle activity.

## 5. Conclusions

Given the highly significant results from application of IE to EEG, with the ability to discriminate between waking and sleep consciousness stages via multiple distinct statistical descriptions, the study of EEG-based information transfer constant (*κ*) certainly deserves to be tried more generally with other sleep EEG datasets to ensure replicability. The application of IE to EEG is very straightforward, with extremely simple programming algorithms compared to other techniques. Indeed, if the results of the present study are a guide, it may be interesting to apply IE more widely with states of brain dysfunction to see if it will become a useful tool in the quantitative analysis of EEG.

## Figures and Tables

**Table 1:** Mean information transfer ratio comparisons.

Δ*T*	Waking versus	Sleep stage 2	*F*_(1,221)_	Sleep stage 3	*F*_(1,184)_
**0.004**	1.08 (0.097)	1.21 (0.08)	170.8^*∗∗∗*^	1.17 (0.08)	144.1^*∗∗∗*^
**0.04**	1.60 (0.18)	1.65 (0.12)	19.8^*∗∗∗*^	1.57 (0.14)	1.67
**0.4**	5.51 (0.48)	5.31 (0.47)	19.1^*∗∗∗*^	4.51 (0.57)	263.1^*∗∗∗*^
**4**	−3.62 (0.33)	−3.53 (0.31)	9.6^*∗∗*^	−3.01 (0.36)	280.1^*∗∗∗*^

Δ*T*: time difference for voltage change calculations, in seconds.

Values represent mean (s.d.) for group mean information transfer ratio (*κ*).

^*∗∗∗*^ *p* < 0.0001; ^*∗∗*^ *p* < 0.001 after Bonferroni correction.

**Table 2 tab2:** Fraction of values with kappa < 0.2 at Δ*T* = 0.004 s.

Waking versus	Sleep stage 2	*F*_(1,209)_
0.018 (0.020)	0.031 (0.023)	753.2^*∗∗∗*^

	Sleep stage 3	*F*_(1,184)_

0.018 (0.020)	0.16 (0.12)	822^*∗∗∗*^

Data represent mean (s.d.) for fraction of *κ* values < 0.2.

Per segment, via repeated-measures ANOVA. ^*∗∗∗*^ *p* < 0.0001.

**Table 3 tab3:** Mean *p* ≥ 0.01 Lomb-Scargle power comparisons.

Δ*T* (sec)	Waking	Sleep stage 2	*F*_(1,209)_	Sleep stage 3	*F*_(1,184)_
**0.004**	1.2 × 10^5^ (2.2 × 10^4^)	1.5 × 10^5^ (2.7 × 10^4^)	78.7^*∗∗∗*^	9.1 × 10^4^ (5.8 × 10^4^)	62.6^*∗∗∗*^
**0.04**	9.8 × 10^4^ (2.2 × 10^4^)	1.0 × 10^5^ (2.2 × 10^4^)	11.87^*∗∗*^	1.5 × 10^5^ (4.2 × 10^4^)	244.4^*∗∗∗*^
**0.4**	3.5 × 10^4^ (6.7 × 10^3^)	3.3 × 10^4^ (5.1 × 10^3^)	11.08^*∗∗*^	4.8 × 10^4^ (1.6 × 10^4^)	100.9^*∗∗∗*^
**4**	5.2 × 10^4^ (9.5 × 10^3^)	5.0 × 10^4^ (7.6 × 10^3^)	6.9^*∗∗*^	7.3 × 10^4^ (2.4 × 10^3^)	119.2^*∗∗∗*^

Listed values represent mean (s.d.) of *p* ≤ 0.01 Lomb-Scargle periodogram power for each state of consciousness. Comparisons were done by repeated-measures ANOVA. ^*∗∗∗*^ *p* < 0.0001; ^*∗∗*^ *p* < 0.01.

**Table 4:** Mean *κ* and LS power for sleep stage 1 and REM sleep versus waking EEG.

Δ*T* (sec)	Waking	Sleep stage 1	*t* stat	REM sleep	*t* stat
Mean *κ* ^a^					
**0.004**	1.06 (0.09)	1.17 (0.1)	2.46^*∗*^	1.27 (0.09)	5.61^*∗∗∗*^
**0.04**	1.56 (0.19)	1.69 (0.08)	**2.04**	1.75 (0.16)	4.15^**∗∗**^
**0.4**	5.39 (0.54)	5.69 (0.42)	**2.42**	5.74 (0.59)	**2.31**
**4**	−3.45 (0.37)	−3.62 (0.23)	1.4	−3.66 (0.41)	1.54

LS power^b^					
**0.004**	7879 (4989)	7766 (4826)	0.05	8422 (9083)	0.18
**0.04**	1.7 × 10^5^ (5.2 × 10^4^)	1.9 × 10^5^ (3.7 × 10^4^)	0.93	2.1 × 10^5^ (6.2 × 10^4^)	3.26^**∗**^
**0.4**	2.9 × 10^5^ (8.3 × 10^4^)	3.7 × 10^5^ (9.4 × 10^4^)	**2.12**	4.0 × 10^5^ (1.3 × 10^5^)	3.32^**∗**^
**4**	4.5 × 10^5^ (1.3 × 10^5^)	6.2 × 10^5^ (1.9 × 10^5^)	2.63^**∗**^	6.3 × 10^5^ (2.0 × 10^5^)	2.76^**∗**^

^a^Listed values represent mean (s.d.) of *κ* values for each state.

^b^Listed values represent mean (s.d.) of *p* ≤ 0.01 Lomb-Scargle periodogram power for each state.

Comparisons were done by GLMM. ^*∗∗∗*^ *p* < 0.001; ^*∗∗*^ *p* < 0.01; ^*∗*^ *p* < 0.05; bold: *p* < 0.1.

## Abbreviations

IE: Information equilibrium.
